# Adaptation and validation of the evidence-based practice profile (EBP^2^) questionnaire in a Norwegian primary healthcare setting

**DOI:** 10.1186/s12909-024-05842-z

**Published:** 2024-08-06

**Authors:** Nils Gunnar Landsverk, Nina Rydland Olsen, Kristine Berg Titlestad, Are Hugo Pripp, Therese Brovold

**Affiliations:** 1 Department of Rehabilitation Science and Health Technology, Faculty of Health Science, Oslo Metropolitan University, Oslo, Norway; 2 Department of Health and Functioning, Faculty of Health and Social Sciences, Western Norway University of Applied Sciences, Bergen, Norway; 3 Faculty of Health Sciences, OsloMet - Oslo Metropolitan University, Oslo, Norway; 4 Department of Welfare and Participation, Faculty of Health and Social Sciences, Western Norway University of Applied Sciences, Bergen, Norway

**Keywords:** Evidence-based practice, Healthcare professional, Primary healthcare, Content validity, Construct validity, Structural validity, Internal consistency, Knowledge, Attitudes, Self-efficacy, Behavior

## Abstract

**Background:**

Access to valid and reliable instruments is essential in the field of implementation science, where the measurement of factors associated with healthcare professionals’ uptake of EBP is central. The Norwegian version of the Evidence-based practice profile questionnaire (EBP^2^-N) measures EBP constructs, such as EBP knowledge, confidence, attitudes, and behavior. Despite its potential utility, the EBP^2^-N requires further validation before being used in a cross-sectional survey targeting different healthcare professionals in Norwegian primary healthcare. This study assessed the content validity, construct validity, and internal consistency of the EBP^2^-N among Norwegian primary healthcare professionals.

**Methods:**

To evaluate the content validity of the EBP^2^-N, we conducted qualitative individual interviews with eight healthcare professionals in primary healthcare from different disciplines. Qualitative data was analyzed using the “text summary” model, followed by panel group discussions, minor linguistic changes, and a pilot test of the revised version. To evaluate construct validity (structural validity) and internal consistency, we used data from a web-based cross-sectional survey among nurses, assistant nurses, physical therapists, occupational therapists, medical doctors, and other professionals (*n* = 313). Structural validity was tested using a confirmatory factor analysis (CFA) on the original five-factor structure, and Cronbach’s alpha was calculated to assess internal consistency.

**Results:**

The qualitative interviews with primary healthcare professionals indicated that the content of the EBP^2^-N was perceived to reflect the constructs intended to be measured by the instrument. However, interviews revealed concerns regarding the formulation of some items, leading to minor linguistic revisions. In addition, several participants expressed that some of the most specific research terms in the terminology domain felt less relevant to them in clinical practice. CFA results exposed partial alignment with the original five-factor model, with the following model fit indices: CFI = 0.749, RMSEA = 0.074, and SRMR = 0.075. Cronbach’s alphas ranged between 0.82 and 0.95 for all domains except for the Sympathy domain (0.69), indicating good internal consistency in four out of five domains.

**Conclusion:**

The EBP^2^-N is a suitable instrument for measuring Norwegian primary healthcare professionals’ EBP knowledge, attitudes, confidence, and behavior. Although EBP^2^-N seems to be an adequate instrument in its current form, we recommend that future research focuses on further assessing the factor structure, evaluating the relevance of the items, and the number of items needed.

**Registration:**

Retrospectively registered (prior to data analysis) in OSF Preregistration. Registration DOI: 10.17605/OSF.IO/428RP.

**Supplementary Information:**

The online version contains supplementary material available at 10.1186/s12909-024-05842-z.

## Background

Evidence-based practice (EBP) integrates the best available research evidence with clinical expertise, patient characteristics, and preferences [1]. The process of EBP is often described as following the five steps: ask, search, appraise, integrate, and evaluate [1, 2]. Practicing the steps of EBP requires that healthcare professionals hold a set of core competencies [3, 4]. Lack of competencies such as EBP knowledge and skills, as well as negative attitudes towards EBP and low self-efficacy, may hinder the implementation of EBP in clinical practice [5–10]. Measuring of EBP competencies may assist organizations in defining performance expectations and directing professional practice toward evidence-based clinical decision-making [11].

Using well-designed and appropriate measurement instruments in healthcare research is fundamental for gathering precise and pertinent data [12, p. 1]. Access to valid and reliable instruments is also essential in the field of implementation science, where conducting consistent measurements of factors associated with healthcare professionals’ uptake of EBP is central [13]. Instruments measuring the uptake of EBP should be comprehensive and reflect the multidimensionality of EBP; they should be valid, reliable, and suitable for the population and setting in which it is to be used [14]. Many instruments measuring different EBP constructs are available today [15–22]. However, the quality of these instruments varies, and rigorous validation studies that aim to build upon and further develop existing EBP instruments are necessary [13, 16].

The authors of this study conducted a systematic review to summarize the measurement properties of existing instruments measuring healthcare professionals’ EBP attitudes, self-efficacy, and behavior [16]. This review identified 34 instruments, five of which were translated into Norwegian [23–27]. Of these five instruments, only the Evidence-based practice profile questionnaire (EBP^2^) was developed to measure various EBP constructs, such as EBP knowledge, confidence, attitudes, and behavior [28]. In addition, EBP^2^ was developed to be trans-professional [28]. Although not exclusively demonstrating high-quality evidence for all measurement properties, the review authors concluded that the EBP^2^ was among the instruments that could be recommended for further use and adaption for use among different healthcare disciplines [16].

EBP^2^ was initially developed by McEvoy et al. in 2010 and validated for Australian academics, practitioners, and students from different professions (physiotherapy, podiatry, occupational therapy, medical radiation, nursing, human movement) [28]. The instrument was later translated into Chinese and Polish and further tested among healthcare professionals in these countries [29–32]. The instrument was also translated into Norwegian and cross-culturally adapted into Norwegian [27]. The authors assessed content validity, face validity, internal consistency, test-retest reliability, measurement error, discriminative validity, and structural validity among bachelor students from nursing and social education and health and social workers from a local hospital [27]. Although the authors established the content validity of the EBP^2^-Norwegian version (EBP^2^-N), they recommended further linguistic improvements. Additionally, while they found the EBP^2^-N valid and reliable for three subscales, the original five-factor model could not be confirmed using confirmatory factor analysis. Therefore, they recommended further research on the instrument measurement properties [27].

We recognized the need for further assessment of measurement properties of the EBP^2^-N before using this instrument in a planned cross-sectional survey targeting physical therapists, occupational therapists, nurses, assistant nurses, and medical doctors working with older people in Norwegian primary healthcare [33]. As our target population differed from the population studied by Titlestad et al. [27], the EBP^2^-N should be validated again, assessing content validity, construct validity and internal consistency [12, p. 152]. The assessment of content validity evaluates whether the content of an instrument is relevant, comprehensive, and understandable for a specific population [34]. Construct validity, including structural validity and cross-cultural validity, can provide evidence on whether an instrument measures what it intends to do [12, p. 169]. Furthermore, the degree of interrelatedness among the items (internal consistency) should be assessed when evaluating how items of a scale are combined [35]. Our objectives were to comprehensively assess content validity, structural validity, and internal consistency of the EBP^2^-N among Norwegian primary healthcare professionals. We hypothesized that the EBP^2^-N was a valid and reliable instrument suitable for use in Norwegian primary healthcare settings.

## Methods

### Study design

This study was conducted in two phases: **Phase 1** comprised a qualitative assessment of the content validity of the EBP^2^-N, followed by minor linguistic adaptions and a pilot test of the adapted version. **Phase 2** comprised an assessment of structural validity and internal consistency of the EBP^2^-N based on the result from a web-based cross-sectional survey.

The design and execution of this study adhered to the COSMIN Study Design checklist for patient-reported outcome measurement instruments, as well as the methodology for assessing the content validity of self-reported outcome measures [34, 36, 37]. Furthermore, this paper was guided by the COSMIN Reporting guidelines for studies on measurement properties of patient-reported outcome measures [38].

### Participants and setting

Participants eligible for inclusion in both phases of this study were health personnel working with older people in primary healthcare in Norway, such as physical therapists, occupational therapists, nurses, assistant nurses, and medical doctors. Proficiency in reading and understanding Norwegian was a prerequisite for inclusion. This study is part of a project called FALLPREVENT, a research project that aims to bridge the gap between research and practice in fall prevention in Norway [39].

### Instrument administration

The EBP^2^-N consists of 58 self-reported items that are divided into five different domains: (1) *Relevance* (items 1–14), which refers to the value, emphasis, and importance respondents place on EBP; (2) *Sympathy* (items 15–21) which refers to the perceived compatibility of EBP with professional work; (3) *Terminology* (items 22–38), which refers to the understanding of common research terms; (4) *Practice* (items 39–47), which refers to the use of EBP in clinical practice and; (5) *Confidence* (items 48–58), which relates to respondents perception of their EBP skills [28]. All the items are rated on a five-point Likert scale (1 to 5) (see questionnaire in Additional file 1). Each domain is summarized, with higher scores indicating a higher degree of the construct measured in the domain in question. The items in the *Sympathy* domain are negatively phrased and need to be reversed before being summarized. The possible range in summarized scores (min-max) per domain are as follows: *Relevance* (14–70), *Sympathy (7-35)*, *Terminology* (17–85), *Practice (9-45)*, and *Confidence* (11–55).

### Phase 1: content validity assessment

#### Recruitment and participant characteristics

Snowball sampling was used to recruit participants in Eastern Norway, and possible eligible participants were contacted via managers in healthcare settings. The number of participants needed for the qualitative content validity interviews was based on the COSMIN methodology recommendations and was set to at least seven participants [34, 37]. We recruited and included eight participants. All participants worked with older people in primary healthcare, and included two physical therapists, two occupational therapists, two assistant nurses, one nurse, and one medical doctor. The median age (min-max) was 35 (28–55). Two participants held upper secondary education, four held a bachelor’s degree, and two held a master’s degree. Six participants reported that they had some EBP training from their education or had attended EBP courses, and two had no EBP training.

#### Qualitative interviews

Before the interviews, a panel of four members (NGL, TB, NRO, and KBT) developed a semi-structured interview guide. Two panel members were EBP experts with extensive experience in EBP research and measurement (NRO and KBT). KBT obtained consent from the developer of the original EBP^2^ questionnaire and translated the questionnaire into Norwegian in 2013 [27].

To evaluate the content validity of the EBP^2^-N for use among different healthcare professionals working in primary healthcare in Norway, we conducted individual interviews with eight healthcare professionals from different disciplines. Topics in the interview guide were guided by the standards of the COSMIN study design checklist and COSMIN criteria for good content validity, which include questions related to the following three aspects [34, 37]: Whether the items of the instrument were perceived relevant (relevance), whether all key concepts were included (comprehensiveness), and whether the instructions, items, and response options were understandable (comprehensibility) [34]. The interview guide is presented in Additional File 2. Interview preparations and training included a review of the interview guide and a pilot interview with a physical therapist not included in the study.

Eight interviews were conducted by the first author (NGL) in May and June 2022. All interviews were conducted in the participant’s workplaces. The interviews followed a “think-aloud” method [12, p. 58, 40, p. 5]. Hence, in the first part of the interview, the participants were asked to complete the questionnaire on paper while simultaneously saying aloud what they were thinking while responding to the questionnaire. Participants also had to state their choice of answer aloud and make a pen mark on the items or responses that either were difficult to understand or did not feel relevant to them. In the second part of the interviews, participants were asked to elaborate on why items were marked as difficult to understand or irrelevant, focusing on relevance and comprehensibility. In addition, the participants were asked to give their overall impression of the instrument and state if they thought any essential items (comprehensiveness) were missing. Only the second part of the interviews were audio-recorded.

#### Analysis and panel group meetings

After conducting the individual interviews, the first author immediately transcribed the recorded audio data. The subsequent step involved gathering and summarizing participants’ comments into one document that comprised the questionnaire instructions, items, and response options. Using the “text summary” model [41, p.61], we summarized the primary “themes” and “problems” identified by participants during the interviews. These were then aligned with the specific item or section of the questionnaire to which the comments were related. For example, comments on the items’ comprehensibility were identified as one “theme”, and the corresponding “problem” was that the item was perceived as too academically formulated or too complex to understand. Comments on an item’s relevance was another “theme” identified, and an example of a corresponding “problem” was that the EBP activity presented in the item was not recognized as usual practice for the participant. The document contained these specific comments and summarized the participants’ overall impression of the instrument. Additionally, it included more general comments addressing the instrument’s relevance, comprehensibility, and comprehensiveness.

Next, multiple rounds of panel group discussions took place, and the final document with a summary of participants’ comments served as the foundation for these discussions. The content validity of the items, instructions, and response options underwent thorough examinations by the panel members. Panel members discussed aspects, such as relevance, comprehensiveness, and comprehensibility, drawing upon insights from interview participants’ comments and the panel members’ extensive knowledge about EBP.

#### Pilot test

Finally, the revised questionnaire was pilot tested on 40 master’s students (physical therapists) to evaluate the time used to respond, and the students were invited to make comments in free text adjacent to each domain in the questionnaire. The pilot participants answered a web-based version of the questionnaire.

### Phase 2: Assessment of structural validity and internal consistency

#### Recruitment and data collection for the cross-sectional survey

Snowball sampling was used to recruit participants. The invitation letter, with information about the study and consent form, was distributed via e-mail to healthcare managers in over 37 cities and municipalities representing the eastern, western, central, and northern parts of Norway. The managers forwarded the invitation to eligible employees and encouraged them to respond to the questionnaire. The respondents that consented to participation automatically received a link to the online survey. Our approach to recruitment made it impossible to keep track of the exact number of potential participants who received invitations to participate. As such, we were unable to determine a response rate.

### Statistical methods

Statistical analyses were performed using STATA [42]. We tested the structural validity and internal consistency of the 58 domain items of the EBP^2^-N, using the same factor structure as in the initial evaluation [28] and the study that translated the questionnaire into Norwegian [27]. Structural validity was assessed using confirmatory factor analysis with maximum likelihood estimation to test if the data fit the predetermined original five-factor structure. Model fit was assessed by evaluating the comparative fit index (CFI), root mean square error of approximation (RMSEA), and the standardized root mean square residual (SRMR). Guidelines suggest that a good-fitting model should have a CFI of around 0.95 or higher, RMSEA of around 0.06 or lower, and SRMR of around 0.08 or lower [43]. Cronbach’s alpha was calculated for each of the five domains to evaluate whether the items within the domains were interrelated. It has been proposed that Cronbach’s alpha between 0.70 and 0.95 can be considered good [44].

The sample size required for a factor analysis was set based on COSMIN criteria for at least an “adequate” sample size, which is at least five times the number of items and > 100 [45, 46]. Accordingly, the sample size required in our case was > 290 respondents. Regarding missing data, respondents with over 25% missing items on domain items were excluded from further analysis. Respondents with over 20% missing on one domain were excluded from the analysis of that domain. The Little’s MCAR test was conducted to test whether data were missing completely at random. Finally, for respondents with 20% or less missing data on one domain, the missing values were substituted with the respondent’s mean of other items within the same domain.

### Ethical approval and consent to participate

The Norwegian Agency for Shared Services in Education and Research (SIKT) approved the study in March 2022 (ref: 747319). We obtained written informed consent from the participants interviewed and the cross-sectional survey participants.

## Results

The findings for Phase 1 and Phase 2 will be presented separately. **Phase 1** will encompass the results of the qualitative content validity assessment, adaptions, and pilot testing of the EBP^2^-N. **Phase 2** will encompass the results of assessing the structural validity and internal consistency of the EBP^2^-N.

### Phase 1: Results of the content validity assessment

#### Comprehensiveness: whether key concepts are missing

Only a few comments were made on comprehensiveness. Notably, one participant expressed the need for additional items addressing clinical experience and user perspectives.

#### Relevance: whether the items are perceived relevant

Overall, the participants commented that they perceived the instrument as relevant to their context. However, several participants pointed out some items that felt less relevant. The *terminology* domain emerged as a specific area of concern, as most participants expressed that this subscale contained items that felt irrelevant to clinical practice. Comments such as “I do not feel it’s necessary to know all these terms to work evidence-based,” and “The more overarching terms like RCT, systematic review, clinical relevance, and meta-analysis I find relevant, but not the more specific statistical terms,” captured the participants’ perspectives on the relevance of the *terminology* domain.

Other comments related to the *terminology* domain revealed that these items could cause feelings of demotivation or inadequacy: “One can become demotivated or feel stupid because of these questions” and “Many will likely choose not to answer the rest of the form, as they would feel embarrassed not knowing”. Other comments on relevance were related to items in other subscales, for example, critical appraisal items (i.e., items 20, 42, and 55), which were considered less relevant by some participants. One participant commented: “If one follows a guideline as recommended, there is no need for critical assessment”.

#### Comprehensibility: Whether instructions, items, and response options are understandable

All eight participants stated that they understood what the term EBP meant. The predominant theme from the participant’s comments was related to the comprehensibility of the EBP^2^-N. Most of the comments on comprehensibility revolved around the formulation of items. Participants noted challenges related to comprehensibility in 35 out of 58 items, either due to difficulty in understanding, readability issues, the length of items, lack of clarity, or overly academic language. For instance, item five in the *Relevance* domain, “I intend to develop knowledge about EBP”, received comments that expressed uncertainty about whether “EBP” referred to the five steps of EBP or evidence-based clinical interventions/practices (e.g., practices following recommendations in evidence-based guidelines). Items that were perceived as overly academic included phrases such as “intend to apply”, “intend to develop”, or “convert your information needs”. For these phrases, participants suggested simpler formulations in layperson’s Norwegian. Some participants deemed the instrument “too advanced,” “on a too high level,” or “too abstract”, and others expressed that they understood most of the instrument’s content, indicating a divergence among participants.

Examples of items considered challenging to read, too complex, or overly lengthy were items six and 12 in the *relevance* domain, 16 and 20 in the *sympathy* domain, and 58 in the *confidence* domain. The typical comments from participants revealed a preference for shorter, less complex items with a clear and singular focus. In addition, some comments referred to the formulation of response options. For instance, two response options in the *confidence* domain, “Reasonably confident” and “Quite confident”, were perceived as too similar in Norwegian. In the practice subscale, a participant pointed out that the term “monthly or less” lacked precision, as it could cover any frequency from once to twelve times a year, thus being perceived as imprecise.

#### Panel group meetings and instrument revision

The results of the interviews were discussed during several rounds of panel group meetings. After thoroughly examining the comments, 33 items underwent revisions during the panel meetings. These revisions primarily involved minor linguistic adjustments to preserve the original meaning of the items. For example, the Norwegian version of item 8 was considered complex and overly academically formulated and underwent revision. The phrase “I intend to apply” was replaced by “I want to use”, as the panel group considered this phrase easier to understand in Norwegian. Another example involved the term “Framework,” which some participants found vague or difficult to understand (i.e., in item 3, “my profession uses EBP as a framework”). The term “framework” was replaced with “way of thinking and working”, considered more concrete and understandable in Norwegian. The phrase “way of thinking and working” was also added to item 5 to clarify that “EBP” referred to the five steps of EBP, not interventions in line with evidence-based recommendations. Additionally, it was challenging to revise items that participants considered challenging to read, too complex, or overly lengthy (i.e., 6, 12, 16, 20, and 58), as it was difficult to shorten them without losing their original meaning. However, replacing overly academic words with simpler formulations made these examples less complex and more readable.

In terms of relevance of the items, no items were removed, and the *terminology* domain was retained despite comments regarding its relevance. Changing this domain would have impeded the opportunity to compare results from future studies using this questionnaire with previous studies using the same questionnaire. Regarding comprehensiveness, the panel group reached a consensus that the domains included all essential items concerning the constructs that the original instrument states to measure. Further, examples of minor linguistic changes and additional details on item revisions are reported in Additional File 3.

#### Pilot test

The median time to answer the questionnaire was nine minutes. Students made no further comments to the questionnaire.

### Phase 2: Assessment of structural validity and internal consistency

#### Participants’ characteristics and mean domain scores

A total of 313 responded to the survey. The respondents’ mean age (SD) was 42.7 years (11.4).The sample included 119 nurses, 74 assistant nurses, 64 physical therapists, 38 occupational therapists, three medical doctors, and 15 other professionals, mainly social educators. In total, 63.9% (*n* = 200) of the participants held a bachelor’s degree, 11.8% (*n* = 37) held a master’s degree, and 0.3% (*n* = 1) held a Ph.D. Moreover, 10.5% (*n* = 33) of the participants had completed upper secondary education, and 13.1% (*n* = 41) had tertiary vocational education. One hundred and eighty-five participants (59.1%) reported no formal EBP training, while among the 128 participants who had undergone formal EBP training, 31.5% had completed over 20 h of EBP training. The mean scores (SD) for the different domains were as follows: *Relevance* 80.2 (7.3), *Sympathy* 21.2 (3.6), *Terminology* 44.5 (15.3), *Practice* 22.2 (5.8), and *Confidence* 31.2 (9.2).

#### Missing data

Out of 314 respondents, one was excluded due to over 25% missing domain items, and three were excluded due to more than 20% missing data in specific domains. Twenty-six respondents had under 20% missing data on one domain, and these missing values were substituted with the respondent’s mean of the other items within the same domain. In total, 313 responses were included in the final analysis. Each domain item had at most 1.3% missing items in total. The percentage of missing data per domain was low and relatively similar across the five domains (*Relevance* = 0.05%, *Sympathy* = 0.2%, *Terminology* = 0.4%, *Practice* = 0.6%, *Confidence* = 0.6%). The Little’s MCAR test showed p-values higher than 0.05 for all domains, indicating that data was missing completely at random.

#### Structural validity results

**Fig. 1:**
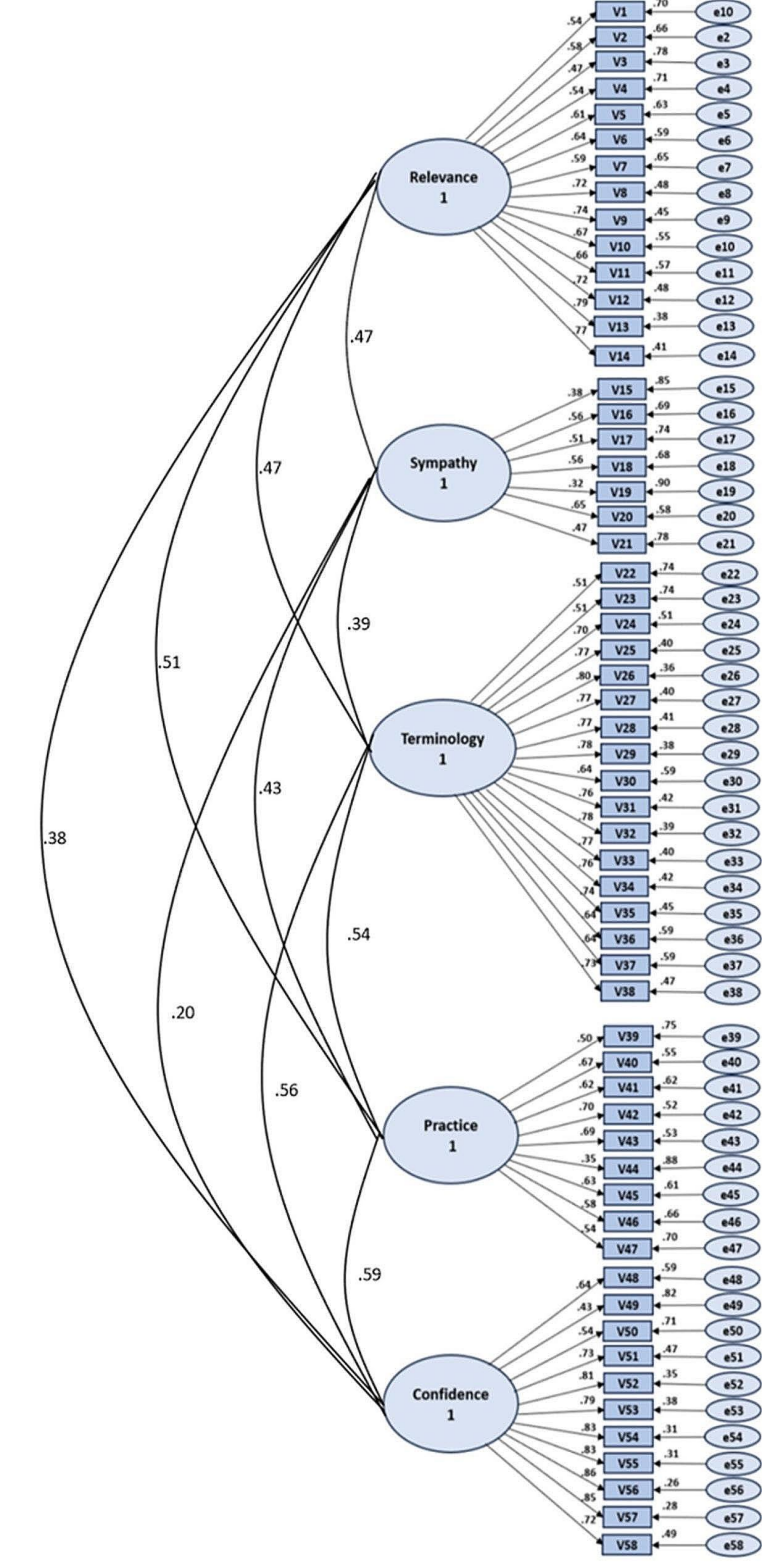
Confirmatory factor analysis, standardized solution of the EBP2-N. (*n* = 313). Note: Large circles = latent variables, Rectangles = measured items, small circles = residual variance

A five-factor model was estimated based on the original five-factor structure (Fig. 1). The model was estimated using the maximum likelihood method. A standardized solution was estimated, constraining the variance of latent variables to 1. Correlation among latent variables was allowed. The results of the CFA showed the following model fit indices: CFI = 0.749, RMSEA = 0.074, and SRMR = 0.075. The CFI and RMSEA results did not meet the criteria for a good-fitting model set a priori (CFI of around 0.95 or higher, RMSEA of around 0.06 or lower). However, the SRMR value met the criteria around 0.08 or lower. All standardized factor loadings were over 0.32, and only five items loaded under 0.5. The range of standardized factor loadings was the following in the different domains: *Relevance* = 0.47–0.79; *Terminology* = 0.51–0.80; *Practice* = 0.35–0.70, *Confidence* = 0.43–0.86, and *Sympathy* = 0.32–0.65 (Fig. 1).

#### Internal consistency results

As reported in Table 1, Cronbach’s alphas ranged between 0.82 and 0.95 for all domains except for the *Sympathy* domain, where Cronbach’s alpha was 0.69. Results indicate good internal consistency for four domains and close to the cut-off of good internal consistency (> 0.70) on *Sympathy.*

**Table 1:** Internal consistency per domain

Domain	No. of items	*n*	Cronbach’s alpha
Relevance	14	313	0.90
Terminology	17	313	0.95
Practice	9	312	0.82
Confidence	11	311	0.93
Sympathy	7	313	0.69

## Discussion

In this study, we aimed to assess the measurement properties of the EBP^2^-N questionnaire. The study population of interest was healthcare professionals working with older people in Norwegian primary healthcare, including physical therapists, occupational therapists, nurses, assistant nurses, and medical doctors. The study was conducted in two phases: content validity was assessed in Phase 1, and construct validity and internal consistency were assessed in phase 2.

The findings from Phase 1 and the qualitative interviews with primary healthcare professionals indicated that the content of the EBP^2^-N was perceived to reflect the constructs intended to be measured by the instrument [28]. However, the interviews also revealed different perceptions regarding the relevance and comprehensibility of certain items. Participants expressed concerns about the formulation of some items, and we decided to make minor linguistic adjustments, aligning with previous recommendations to refine item wording through interviews [27]. Lack of content validity can have adverse consequences [34]. Irrelevant or incomprehensible items may make respondents tired of answering, leading to potentially biased answers [47, 48, p. 139]. Analysis of missing data showed that possible irrelevant or incomprehensible items did not lead to respondent fatigue, as the overall percentage of missing items was low (at most 1.3%), and the percentage of missing data did not vary across the domains. Irrelevant items may also impact other measurement properties, such as structural validity and internal consistency [34]. We believe that the minor linguistic revisions we made to some items made the questionnaire easier to understand. This assumption was supported by the pilot test of 40 master’s students, where no further comments regarding comprehensibility were added.

The overall relevance of the instruments was perceived positively. However, several participants expressed concerns about the terminology domain as some of the most specific research terms felt irrelevant to them in clinical practice. Still, the panel group decided to keep all items in the terminology domain to allow comparison of results among future studies on the same instrument and subscales. In addition, this decision was based on the fact that knowledge about research terminology, such as “types of data,” “measures of effect,” and “statistical significance,” are essential competencies to perform step three of the EBP process (critical appraisal) [3]. Leaving out parts of the terminology domain could, therefore, possibly make our assessment of the EBP constructs less comprehensive and complete [14]. However, since the relevance of some items in the terminology domain was questioned, we cannot fully confirm the content validity of this domain, and we recommend interpreting it with caution.

The confirmatory factor analysis (CFA) in Phase 2 of this study revealed that the five-factor model only partially reflected the dimensionality of the constructs measured by the instrument. The SRMR was the only model fit indices that completely met the criteria for a good-fitting model set a priori, yielding a value of 0.075. In contrast, the CFI at 0.749 and RMSEA at 0.074 fell short of the criteria for a good-fitting model (CFI ≥ 0.95, RMSEA ≤ 0.06). However, our model fit indices were closer to the criteria for a good-fitting model compared to Titlestad et al. (2017) [27] who demonstrated a CFI of 0.69, RMSEA of 0.089, and SRMR of 0.095. This tendency toward better fit in our study may be related to the larger sample size, in agreement with established recommendations of a minimum of 100–200 participants and at least 5–10 times the number of items to ensure the precision of the model and overall model fit [46, p. 380].

Although our sample size met COSMIN’s criteria for an “adequate” sample size [45], the partially adequate fit indices suggest that the original five-factor model might not be the best-fitting model. A recent study on the Chinese adaptation of the EBP^2^ demonstrated that item reduction and using a four-factor structure improved model fit (RMSEA = 0.052, CFI = 0.932) [30]. The same study removed eighteen items based on content validity evaluation (four from *relevance*, seven from *terminology*, and seven from *sympathy*) [30]. In another study where the EBP^2^ was adapted for use among Chinese nurses, thirteen items (two from *sympathy*, eight from *terminology*, one from *practice*, and two from *confidence*) were removed, and an eight-factor structure was identified [29]. However, compared to our study, noticeably improved model fit was not demonstrated in this study [29]. The model fit indices of their 45-item eight-factor structure were quite similar to the one found in our study (RMSEA = 0.065, SRMR = 0.077, CFI = 0.884) [29]. The results from the two above mentioned studies suggest that a model including fewer items and another factor structure potentially could have applied to our population as well. Although the five-factor model only partially reflects the constructs measured by the EBP^2^-N in our population, it contributes valuable insights into the instrument’s performance in a specific healthcare setting.

Cronbach’s alpha results in this study indicate good internal consistency for four domains, being over 0.82. However, the alpha of 0.69 in the sympathy did not reach the pre-specified cut-off of good internal consistency (0.70) [44]. A tendency of relatively lower Cronbach’s alpha values on the sympathy domain, compared to the other four domains, has also been identified in previous similar studies [27, 28, 31, 32]. Titlestad et al. (2017) reported Cronbach’s alpha to be 0.66 in the sympathy domain and above 0.90 in the other domains [27]. McEvoy et al. (2010), Panczyk et al. (2017), and Belowska et al. (2020) reported Cronbach’s alphas of 0.76–0.80 for the sympathy domain, and 0.85–0.97 for the other domains [28, 31, 32]. In these three cases, Cronbach’s alphas of the sympathy domain were all over 0.70, but the same tendency of this domain demonstrating lower alphas than the other four domains was evident. The relatively lower alpha values in the sympathy domain may be related to the negative phrasing of items [49], the low number of items in this domain compared to the others (*n* = 7) [12, p. 84, 47, p. 86], and a possible heterogeneity in the construct measured [47, p. 232]. The internal consistency results of our study indicate that the items in the sympathy domain are less interrelated than the other domains. However, having a Cronbach’s alpha value of 0.69 indicates that the items do not entirely lack interrelatedness.

### Limitations

Methodological limitations that could potentially introduce bias into the results should be acknowledged. Although the eight participants involved in the qualitative content validity interviews in Phase 1 covered all healthcare disciplines and education levels aimed to be included in the survey in Phase 2, it remains uncertain whether these eight participants demonstrated all potential variations in the population of interest. It is possible that those that agreed to participate in qualitative interviews regarding an EBP instrument held more positive attitudes toward EBP than the general practitioner would do. Another possible limitation pertains to the qualitative interviews and the fact that the interviewer (NGL) had limited experience facilitating “think-aloud” interviews. To reduce the potential risk of bias related to the interviewer, the panel group with extensive experience in EBP research took part in the interview preparation, and a pilot interview was conducted before the interviews to ensure training.

Furthermore, using a non-random sampling method and the unknown response rate in Phase 2 may have led to biased estimates of measurement properties and affected the representativeness of the sample included. Additionally, the characteristics of non-responders remain unknown, making it challenging to assess whether they differ from the responders and if the final sample adequately represents the variability in the construct of interest. Due to potential selection bias and non-response bias, there may be uncertainty regarding the accuracy of the measurement property assessment and whether the study sample fully represents the entire population of interest [50, p. 205].

## Conclusions

The EBP^2^-N is suitable for measuring Norwegian primary healthcare professionals’ EBP knowledge, attitudes, confidence, and behavior. Researchers can use the EBP^2^-N to increase their understanding of factors affecting healthcare professional’s implementation of EBP and to guide the development of tailored strategies for implementing EBP.

This study revealed positive perceptions of the content validity of the EBP^2^-N, though with nuanced concerns about the relevance and comprehensibility of certain items and uncertainty regarding the five-factor structure of the EBP^2^-N. The minor linguistic revisions we made to some items made the questionnaire more understandable. However, when EBP^2^-N is used in primary healthcare, caution should be exercised when interpreting the results of the terminology domain, as the relevance of some items has been questioned.

Future research should focus on further assessing the factor structure of the EBP^2^-N, evaluating the relevance of the items, and exploring the possibility of reducing the number of items, especially when applied in a new setting or population. Such evaluations could further enhance our understanding of the instrument’s measurement properties and potentially lead to improvements in the measurement properties of the EBP^2^-N.

### Electronic supplementary material

Below is the link to the electronic supplementary material.

**Supplementary Material 1:** The EBP2-N questionnaire

**Supplementary Material 2:** The interview guide

**Supplementary Material 3:** Details on item revisions

**Supplementary Material 4:** Reporting guideline

## Data Availability

The datasets used and analyzed during the current study are available from the corresponding author on reasonable request.

## Abbreviations

EBP: Evidence-based practice
EBP^2^: The Evidence-based practice profile
EBP^2^-N: The Norwegian version of the Evidence-based practice profile questionnaire
COSMIN: Consensus-based Standards for the Selection of Health Measurement Instruments
CFA: Confirmatory factor analysis
CFI: Comparative fit index
RMSEA: Root mean square error of approximation
SRMR: Standardized square residual
SIKT: The Norwegian Agency for Shared Services in Education and Research
