# Supplementary material for: Adaptation and validation of the evidence-based practice profile (EBP2) questionnaire in a Norwegian primary healthcare setting
Source: BMC Med Educ. 2024 Aug 6;24:841. doi: 10.1186/s12909-024-05842-z (PMC11301838; doi:10.1186/s12909-024-05842-z)
Supplement: Supplementary file 1 — Supplementary Material 1: The EBP2-N questionnaire [file 12909_2024_5842_MOESM1_ESM.pdf]

OSLO METROPOLITAN UNIVERSITY
STORBYUNIVERSITETET

Date: _______________

Participant ID nr: ______

Interview guide for assessing the content validity of the Evidence-based practice profile questionnaire (EBP2), Norwegian version

Background variables:

**Age:**	**Education level:**
**EBP-training**	**Profession**
**Time think aloud**	**Time interview:**

Text not read out loud to the person being interviewed is written in green font.

Information about the purpose of the interview:

The Evidence-based practice profile questionnaire (EBP2) is a questionnaire that measures healthcare professionals' knowledge, attitudes, behavior, and confidence related to evidence-based practice. Before this questionnaire is used in a survey, we want healthcare professionals in the target population (Nurses, Physical therapists, Occupational therapists, assistant nurses, and medical doctors) to test it out and provide comments on the questionnaire. We want to examine 1) Comprehensibility (whether questions, response options, and instructions are understandable), 2) Relevance (Whether the questions are perceived as relevant), and 3) Comprehensiveness (if there are missing questions related to what is being measured). The results from this interview will be analyzed and used to make any necessary revisions to the questionnaire before it is used in a survey.

The informant will start by answering all the questions in the questionnaire using the "think aloud" method, followed by the interview.

Think aloud method:

I would now like you to do the following:

Read the instructions and questions aloud, one by one, reflect aloud, and give your answer aloud.

- You may focus on and make a pen mark in the text where you experience any of the following:
    1. Terms/ expressions you do not understand.
    2. Response options that are challenging to understand/ use.
    3. Questions that are hard to read or must be read several times to understand.
    4. Questions that do not make sense.
    5. Questions that ask about multiple things simultaneously, have contradictions or repetitions.
    6. Questions you perceive as irrelevant to you.

(The participant will receive a note with these bullet points, so they don't have to ask the interviewer if they forget them)

The interview (with audio recording):

- I would like to remind you that this interview will be audio-recorded from now on. (Instruct the person being interviewed not to provide information that will increase the likelihood of recognition (e.g., geography, institution the person being interviewed is affiliated with).
- Can you now provide feedback (broadly) on your overall impression of the questionnaire?
- Next, we will go through the questions in the questionnaire where you have marked something (due to comprehensibility or relevance). (Remember to say the number of the question).
- We will summarize each question you commented on together.

If necessary, the interviewer will ask for feedback on additional questions (we are unsure about) and hear what the person being interviewed thinks (given they have yet to talk about it).

Finally, the following questions are asked:

- Are there any questions that you feel are missing in this questionnaire?
- In conclusion, do you have any other comments?
