# Supplementary material for: Adaptation and validation of the evidence-based practice profile (EBP2) questionnaire in a Norwegian primary healthcare setting
Source: BMC Med Educ. 2024 Aug 6;24:841. doi: 10.1186/s12909-024-05842-z (PMC11301838; doi:10.1186/s12909-024-05842-z)
Supplement: Supplementary file 2 — Supplementary Material 2: The interview guide [file 12909_2024_5842_MOESM2_ESM.pdf]

OSLO METROPOLITAN UNIVERSITY
STORBYUNIVERSITETET

HØGSKOLEN I BERGEN

Kunnskapsbasert praksis profil

Målet med dette spørreskjemaet er å samle inn data om kunnskap, atferd og holdninger knyttet til kunnskapsbasert praksis (KBP).

KBP er å ta faglige avgjørelser basert på systematisk innhentet forskningsbasert kunnskap, erfaringsbasert kunnskap og pasientens/brukerens ønsker og behov i en gitt situasjon.

Instruksjoner:

Vi vil være veldig takknemlige hvis du tar deg tid til å besvare dette spørreskjemaet.
Det vil ta ca. 10 minutter å fylle ut skjemaet.
Vennligst sett ring rundt ett tall på hvert spørsmål.
Du kan legge til eventuelle kommentarer til dine svar i feltene: "Har du noen kommentarer til svarene dine?".

Takk for at du tar deg tid til å delta i denne undersøkelsen.

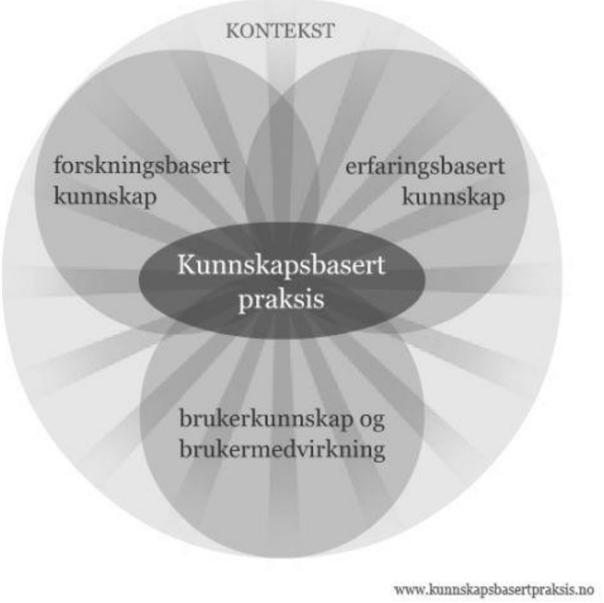

Developed by Maureen Patricia McEvoy, Marie T. Williams & Timothy Stephen Olds, 2010.
Oversatt til norsk av Kristine Berg Titlestad, Høgskolen i Bergen, Avdeling for helse- og sosialfag, Senter for kunnskapsbasert praksis, 2014. Tilpasset av Nils Gunnar Landsverk og Therese Brovold, fakultet for helsevitenskap, OsloMet, og Nina Rydland Olsen, Høgskulen på Vestlandet, institutt for helse og funksjon, 2022.

Grader ditt SVAR på følgende påstander:

	Stemmer ikke i det hele tatt	Stemmer ikke helt	Stemmer delvis	Stemmer mest sannsynlig	Stemmer helt
1. Jeg forstår hva som menes med begrepet kunnskapsbasert praksis (KBP)	1	2	3	4	5
2. Jeg kjenner til KBP innen min profesjon	1	2	3	4	5
3. KBP brukes som tenke- og arbeidsmåte innen min profesjon	1	2	3	4	5
4. Jeg kjenner til pågående utvikling av KBP innen min profesjon	1	2	3	4	5

Har du noen kommentarer til svarene dine?

____

____

____

Grader ditt SVAR på følgende påstander:

	Ingen hensikt i det hele tatt	Usannsynlig å vurdere å gjøre det	Kan vurdere å gjøre det	Vil høyst sannsynlig vurdere å gjøre det	Vil absolutt gjøre det/fortsette å gjøre det
5. Jeg ønsker å lære mer om KBP som tenke- og arbeidsmåte	1	2	3	4	5
6. Jeg ønsker å lære meg å søke etter og kritisk vurdere forskningsbasert kunnskap som er relevant for mitt praksisfelt	1	2	3	4	5
7. Jeg ønsker å lese relevant litteratur for å holde meg oppdatert	1	2	3	4	5
8. Jeg ønsker å bruke forskningsbasert kunnskap for å forbedre praksis	1	2	3	4	5

Har du noen kommentarer til svarene dine?

____

____

____

Grader ditt SVAR på følgende påstander:

	Svært uenig	Uenig	Verken enig eller uenig	Enig	Svært enig
9. Bruk av KBP er nødvendig i mitt arbeid	1	2	3	4	5
10. Litteratur og forskningsfunn er nyttig i mitt daglige arbeid	1	2	3	4	5
11. Det er nødvendig å bruke mer forskningsbasert kunnskap i mitt daglige arbeid	1	2	3	4	5
12. Jeg er interessert i å lære eller forbedre ferdigheter som er nødvendige for å kunne arbeide kunnskapsbasert	1	2	3	4	5
13. KBP forbedrer kvaliteten på mitt arbeid	1	2	3	4	5
14. KBP hjelper meg å ta faglige avgjørelser som omhandler pasienter/brukere i mitt arbeid	1	2	3	4	5
15. KBP tar ikke høyde for de begrensninger (f.eks. tid og utstyr) jeg møter i mitt daglige arbeid	1	2	3	4	5
16. Det er ikke noe poeng i å jobbe kunnskapsbasert fordi det mangler relevant forskningsbasert kunnskap som er til å stole på	1	2	3	4	5
17. KBP tar ikke hensyn til mine pasienters/ brukeres ønsker, behov og verdier	1	2	3	4	5
18. Når jeg tar faglige avgjørelser legger jeg større vekt på egen klinisk erfaring enn på forskningsartikler	1	2	3	4	5
19. Erfaringsbasert kunnskap gir den mest pålitelige kunnskapen om hva som virkelig virker	1	2	3	4	5
20. I min hverdag er det upraktisk å kritisk vurdere kilder til kunnskap (f.eks. oppslagsverk, retningslinjer, systematiske oversikter og enkeltstudier) og vurdere relevans for pasient/ bruker	1	2	3	4	5
21. Å søke etter relevant forskningsbasert kunnskap er ikke så lett å gjennomføre i praksis	1	2	3	4	5

Har du noen kommentarer til svarene dine?

Grader din FORSTÅELSE av følgende begreper:

	Har aldri hørt begrepet	Har hørt det, men forstår det ikke	Har litt forståelse	Forstår det ganske bra	Forstår det og kan forklare det for andre
22. Relative risk/relativ risiko	1	2	3	4	5
23. Absolute risk/absolutt risiko	1	2	3	4	5
24. Systematic review/systematisk oversikt	1	2	3	4	5
25. Odds ratio	1	2	3	4	5
26. Meta analysis/metaanalyse	1	2	3	4	5
27. Number needed to treat	1	2	3	4	5
28. Confidence interval/Konfidensintervall	1	2	3	4	5
29. Publication bias/Publikasjonsskjevhet	1	2	3	4	5
30. Forest plot	1	2	3	4	5
31. Intention to treat	1	2	3	4	5
32. Statistical significance/Statistisk signifikans	1	2	3	4	5
33. Minimum clinically worthwhile effect/ minste klinisk betydningsfulle effekt	1	2	3	4	5
34. Clinical importance/klinisk relevans	1	2	3	4	5
35. Randomised controlled trial (RCT)/ randomisert kontrollert studie	1	2	3	4	5
36. Dichotomous outcomes/dikotome utfall	1	2	3	4	5
37. Continuous outcomes/kontinuerlige utfall	1	2	3	4	5
38. Treatment effect size/behandlingseffektstørrelse	1	2	3	4	5

Har du noen kommentarer til svarene dine?

____

I LØPET AV DET SISTE ÅRET, HVOR OFTE har du?

	Aldri	Månedlig eller sjeldnere	Hver 14. dag	Ukentlig	Daglig
39. Utformet et klart formulert spørsmål hvor pasient/bruker, intervensjon og utfall av interesse er definert	1	2	3	4	5
40. Funnet relevant forskningsbasert kunnskap etter å ha formulert et presist spørsmål	1	2	3	4	5
41. Søkt i elektroniske databaser (som f.eks. MEDLINE) eller kliniske oppslagsverk (som f.eks. UpToDate)	1	2	3	4	5
42. Kritisk vurdert kilder til kunnskap (f.eks. oppslagsverk, retningslinjer, systematiske oversikter og enkeltstudier) du har funnet, for å vurdere metodisk kvalitet	1	2	3	4	5
43. Brukt forskningsbasert kunnskap sammen med egen erfaring for å ta kliniske beslutninger	1	2	3	4	5
44. Tatt hensyn til pasientens/brukerens ønsker, behov og verdier når du har tatt kliniske beslutninger	1	2	3	4	5
45. Lest publiserte forskningsartikler	1	2	3	4	5
46. Uformelt delt og diskutert kilder til kunnskap (f.eks. oppslagsverk, retningslinjer, systematiske oversikter og enkeltstudier) med andre på din arbeidsplass	1	2	3	4	5
47. Formelt delt og diskutert kilder til kunnskap (f.eks. oppslagsverk, retningslinjer, systematiske oversikter og enkeltstudier) med andre på din arbeidsplass (f.eks. Journal club, internundervisning)	1	2	3	4	5

Har du noen kommentarer til svarene dine?

Grader HVOR TRYGG DU FØLER DEG på følgende KBP-aktiviteter:

	Ikke trygg i det hele tatt	Litt trygg	Rimelig trygg	Ganske trygg	Veldig trygg
48. Å forske (forskningsferdigheter)	1	2	3	4	5
49. Å håndtere datamaskiner (dataferdigheter)	1	2	3	4	5
50. Å identifisere hva du mangler kunnskap om i egen praksis	1	2	3	4	5
51. Å omforme kliniske problemstillinger til presise spørsmål som lar seg besvare med forskningsbasert kunnskap	1	2	3	4	5
52. Å ha kjennskap til viktige informasjonskilder (som f.eks.: kliniske oppslagsverk og databaser)	1	2	3	4	5
53. Å søke i en elektronisk database	1	2	3	4	5
54. Å søke etter og skaffe fulltekst av forskningsbasert kunnskap	1	2	3	4	5
55. Å kritisk vurdere forskningsbasert kunnskap etter fastsatte standarder, som for eksempel sjekklister/kvalitetskåringsverktøy for å vurdere metodisk kvalitet	1	2	3	4	5
56. Å vurdere om kilder til kunnskap (oppslagsverk, retningslinjer, systematiske oversikter og enkeltstudier) er til å stole på	1	2	3	4	5
57. Å vurdere om kilder til kunnskap (oppslagsverk, retningslinjer, systematiske oversikter og enkeltstudier) er klinisk anvendbar	1	2	3	4	5
58. Å bruke kilder til kunnskap (oppslagsverk, retningslinjer, systematiske oversikter og enkeltstudier) og tilpasse kunnskapen til den enkelte pasient/ bruker med sine ønsker og behov	1	2	3	4	5

Har du noen kommentarer til svarene dine?

Grader ditt SVAR på følgende påstander:

	Svært uenig	Uenig	Verken enig eller uenig	Enig	Svært enig
59. Jeg setter av tid til å lesekilder til kunnskap som kliniske oppslagsverk, retningslinjer eller forskningsartikler	1	2	3	4	5
60. Jeg har fått opplæring i søk etter retningslinjer og systematiske oversikter	1	2	3	4	5
61. Jeg har fått opplæring i kritisk vurdering av forskningsartikler	1	2	3	4	5
62. For liten tid er en av de viktigste hindringene for å jobbe kunnskapsbasert	1	2	3	4	5
63. Mangel på enkel tilgang til datamaskiner hindrer meg i å praktisere KBP	1	2	3	4	5
64. Støtte fra kollegaer er en av de viktigste faktorene som fremmer om jeg jobber kunnskapsbasert i klinisk praksis	1	2	3	4	5
65. Støtte fra ledelsen er en av de viktigste faktorene som fremmer om jeg jobber kunnskapsbasert i klinisk praksis	1	2	3	4	5
66. Ledelsen ved min arbeidsplass forventer at jeg jobber kunnskapsbasert	1	2	3	4	5

Har du noen kommentarer til svarene dine?

Bakgrunnsvariabler

1. Alder: __________

3. Profesjon (sett kun ett kryss)

- ☐ Ergoterapeut
- ☐ Lege
- ☐ Fysioterapeut
- ☐ Sykepleier
- ☐ Hjelpepleier/ Helsefagarbeider
- ☐ Annet

4. Utdanningsnivå:

- ☐ Grunnskolenivå
- ☐ Videregående skolenivå
- ☐ Fagskolenivå
- ☐ Bachelorgrad
- ☐ Mastergrad
- ☐ Doktorgrad/ PhD

5. Hvor lenge siden er det du var ferdig med siste gjennomførte untdanning?

Antall år_______________

6. Hvor lenge har du jobbet i primærhelsetjenesten ___________

7. Har du fått formell opplæring i KBP? ☐ Ja ☐ Nei

Hvis ja på spørsmål 7, hvor mange timer?

- ☐ 1-3 timer
- ☐ 3-10 timer
- ☐ 10-20 timer
- ☐ Over 20 timer
