# Supplementary material for: Adaptation and validation of the evidence-based practice profile (EBP2) questionnaire in a Norwegian primary healthcare setting
Source: BMC Med Educ. 2024 Aug 6;24:841. doi: 10.1186/s12909-024-05842-z (PMC11301838; doi:10.1186/s12909-024-05842-z)
Supplement: Supplementary file 3 — Supplementary Material 3: Details on item revisions [file 12909_2024_5842_MOESM3_ESM.pdf]

**Additional file 3. Details on item revision**			
**Item**	**Original Version of McEvoy et al. (1)**	**Translated version of Titlestad et al. (2)**	**Revised version of this study**
		**Relevance domain**	
**3**	My profession uses EBP as a framework	KBP brukes som **rammeverk** innen min profesjon	KBP brukes som **tenke- og arbeidsmåte** innen min profesjon
**5**	I intend to develop knowledge about EBP	Jeg **har til hensikt** å tilegne meg kunnskap om KBP	Jeg **ønsker å lære mer om** KBP som **tenke- og arbeidsmåte**
**6**	I intend to develop skills in accessing, acquiring and appraising evidence relevant to my area of practice	Jeg **har til hensikt å tilegne** meg ferdigheter i å søke etter, innhente og kritisk vurdere forskningsbasert kunnskap som er relevant for mitt praksisfelt	Jeg **ønsker å lære** å søke etter og kritisk vurdere forskningsbasert kunnskap som er relevant for mitt praksisfelt
**7**	I intend to read relevant literature to update knowledge	Jeg **har til hensikt** å lese relevant litteratur for å oppdatere min kunnskap	Jeg **ønsker å** lese relevant litteratur for å holde meg oppdatert
**8**	I intend to apply best available evidence findings to improve practice	Jeg **har til hensikt** å anvende beste tilgjengelige forskningsbaserte kunnskap for å forbedre praksis	Jeg **ønsker å** bruke forskningsbasert kunnskap for å forbedre praksis
**9**	Application of EBP is necessary in my work	**Anvendelse** av KBP er nødvendig i mitt arbeid	**Bruk** av KBP er nødvendig i mitt arbeid
**11**	I need to increase the use of evidence in my daily work	**Jeg må øke bruken** av forskningsbasert kunnskap i mitt daglige arbeid	**Det er nødvendig å bruke mer** forskningsbasert kunnskap i mitt daglige arbeid
**12**	I am interested in learning or improving the skills necessary to incorporate EBP into my work	Jeg er interessert i å lære eller forbedre ferdigheter som er nødvendig for å kunne **integrere KBP i mitt arbeid**	Jeg er interessert i å lære eller forbedre ferdigheter som er nødvendige for å kunne **jobbe kunnskapsbasert**
		**Sympathy domain**	
**15**	EBP does not take into account the limitations of my day-to-day work	KBP tar ikke høyde for de begrensninger jeg møter i mitt daglige arbeid	KBP tar ikke høyde for de begrensninger (**f.eks: tid og utstyr**) jeg møter i mitt daglige arbeid
**16**	There isn't much point in doing EBP because there is a lack of strong evidence to support most of the work I do	Det er **har ingen hensikt å utøve KBP** fordi det er mangel på solid forskningsbasert kunnskap som understøtter det meste av arbeidet jeg gjør	Det **er ikke noe poeng i** å **jobbe kunnskapsbasert** fordi det mangler relevant forskningsbasert kunnskap **som er til å stole på**
**17**	EBP does not take into account my clients' preferences	KBP tar ikke hensyn til mine pasienters/brukeres **preferanser**	KBP tar ikke hensyn til mine pasienters/ brukeres **ønsker, behov og verdier**
**18**	In making decisions about my professional work, I value clinical/field experience more than scientific studies	Når jeg tar faglige avgjørelser legger jeg større vekt på **klinisk/praktisk erfaring** enn på vitenskapelige studier	Når jeg tar faglige avgjørelser legger jeg større vekt på **egen klinisk erfaring** enn på forskningsartikler
**20**	Critical appraisal of the literature and its relevance to the client is not very practical in the real world of my profession	Å kritisk vurdere litteratur og dens relevans for pasient/bruker er ikke så lett å gjennomføre i virkeligheten innen min profesjon	I min hverdag er det upraktisk å kritisk vurdere kilder til kunnskap (f**.eks. oppslagsverk, retningslinjer, systematiske oversikter og enkeltstudier**) og vurdere relevans for pasient/ bruker
**21**	Seeking relevant evidence from scientific studies is not very practical in the real world	Å søke etter relevant forskningsbasert kunnskap fra **vitenskapelige studier** er ikke så lett å gjennomføre i praksis	Å søke etter relevant forskningsbasert kunnskap er ikke så lett å gjennomføre i praksis
		**Practice domain**	
**39**	Formulated a clearly answerable question that defines the client or problem, the intervention and outcome(s) of interest	Formulert et **presist** spørsmål som definerer pasient/bruker **eller problem**, intervensjon og utfall av interesse	**Utformet** et **klart** formulert spørsmål hvor pasient/bruker, intervensjon og utfall av interesse er definert
**41**	Searched an electronic database	Søkt i en elektronisk database	Søkt i elektroniske databaser (**som f.eks: medline) eller kliniske oppslagsverk (som f.eks: UpToDate**)
**42**	Critically appraised any literature you have discovered to determine the methodological quality	Kritisk vurdert litteratur du har funnet, for å vurdere metodisk kvalitet	Kritisk vurdert **kilder til kunnskap** (**f.eks. oppslagsverk, retningslinjer, systematiske oversikter og enkeltstudie**r) du har funnet, for å vurdere metodisk kvalitet

**43**	Integrated research evidence with your expertise	Integrert forskningsbasert kunnskap med egen erfaring	Brukt forskningsbasert kunnskap sammen med egen **erfaring for å ta kliniske beslutninger**
**44**	Considered your clients' preferences when making clinical/professional decisions	Tatt hensyn til pasientens/brukerens **preferanser** når du har tatt kliniske/faglige beslutninger	Tatt hensyn til pasientens/brukerens **ønsker, behov og verdier** når du har tatt kliniske beslutninger
**45**	Read published research reports	Lest publiserte forsknings**rapporter**	Lest publiserte forsknings**artikler**
**46**	Informally shared and discussed literature/research findings with others in your workplace	Uformelt delt og diskutert **litteratur/ forskningsfunn** med andre på din arbeidsplass	Uformelt delt og diskutert **kilder til kunnskap (f.eks. oppslagsverk, retningslinjer, systematiske oversikter og enkeltstudier**) med andre på din arbeidsplass
**47**	Formally shared and discussed literature/research findings with others in your department/practice (eg journal club, in-service presentation)	Formelt delt og diskutert **litteratur/ forskningsfunn** med andre på din avdeling/praksis (for eksempel i form av journal club, internundervisning)	Formelt delt og diskutert **kilder til kunnskap (f.eks. oppslagsverk, retningslinjer, systematiske oversikter og enkeltstudier**) med andre på din arbeidsplass (f.eks. Journal club, internundervisning)
		**Confidence domain**	
**48**	Research Skills	Forskningsferdigheter	**Å forske** (forskningsferdigheter)
**49**	Computer skills	Dataferdigheter	**Å håndtere datamaskiner** (dataferdigheter)
**50**	Ability to identify gaps in your knowledge	Evne til å identifisere **egne kunnskapshull**	Å identifisere **hva du mangler kunnskap** om i egen praksis
**51**	Ability to convert your information needs into clearly answerable questions	Evne til å **omsette eget informasjonsbehov** til presise spørsmål som lar seg besvare	Å **omforme kliniske problemstillinger** til presise spørsmål som lar seg besvare med forskningsbasert kunnskap
**52**	Awareness of major information types and sources	Kjennskap til viktige informasjonskilder	Å ha kjennskap til viktige informasjonskilder (**som f.eks: kliniske oppslagsverk og databaser**)
**54**	Ability to access evidence (get copies of articles or reports)	Evne til å innhente forskningsbasert kunnskap (**skaffe kopier av artikler og rapporter**)	Å søke etter og **skaffe fulltekst** av forskningsbasert kunnskap
**55**	Ability to critically analyse evidence against set standards ie quality scoring	Evne til å kritisk vurdere forskningsbasert kunnskap etter fastsatte standarder, som for eksempel sjekklister/kvalitetskåringsverktøy	Å kritisk vurdere forskningsbasert kunnskap etter fastsatte standarder, som for eksempel sjekklister/kvalitetskåringsverktøy **for å vurdere metodisk kvalitet**
**56**	Ability to determine how valid (close to the truth) the material is	Evne til å fastsette hvor **gyldig** (nært opp til sannheten) studien er	Å vurdere om kilder til kunnskap (oppslagsverk, retningslinjer, systematiske oversikter og enkeltstudier) **er til å stole på**
**57**	Ability to determine how useful (clinically applicable) the material is	Evne til å fastsette hvor **nyttig (klinisk anvendbar)** studien er	Å vurdere om kilder til kunnskap (oppslagsverk, retningslinjer, systematiske oversikter og enkeltstudier) **er klinisk anvendbar**
**58**	Ability to apply information to individual cases (ie integrate research evidence with personal preferences, values, concerns, expectations)	Evne til **å anvende** generell informasjon til individuell pasient/bruker i en gitt situasjoner (som for eksempel integrere forskningsbasert kunnskap med personlige **preferanser**, verdier, hensyn og forventninger)	**Å bruke** kilder til kunnskap (oppslagsverk, retningslinjer, systematiske oversikter og enkeltstudier) **og tilpasse kunnskapen** til den enkelte pasient/ bruker med sine **ønsker og behov**

1. McEvoy MP, Williams MT, Olds TS. Development and psychometric testing of a trans-professional evidence-based practice profile questionnaire. Med Teach. 2010;32(9):e373-80.
2. Titlestad KB, Snibsoer AK, Stromme H, Nortvedt MW, Graverholt B, Espehaug B. Translation, cross-cultural adaption and measurement properties of the evidence-based practice profile. BMC Res Notes. 2017;10(1):44.
