# Supplementary material for: Adaptation and validation of the evidence-based practice profile (EBP2) questionnaire in a Norwegian primary healthcare setting
Source: BMC Med Educ. 2024 Aug 6;24:841. doi: 10.1186/s12909-024-05842-z (PMC11301838; doi:10.1186/s12909-024-05842-z)
Supplement: Supplementary file 4 — Supplementary Material 4: Reporting guideline [file 12909_2024_5842_MOESM4_ESM.pdf]

COSMIN Reporting guideline for studies on measurement properties of patient reported outcome measures

Version August 2021

Joel J Gagnier[1-2]
Jianyu Lai[1]
Lidwine B. Mokkink[3]
Caroline B. Terwee[3]

1. Department of Orthopaedic Surgery, University of Michigan, Ann Arbor, MI, USA
2. Department of Epidemiology, School of Public Health, University of Michigan, Ann Arbor, MI, USA
3. Amsterdam UMC, Vrije Universiteit Amsterdam, Department of Epidemiology and Data Science, Amsterdam Public Health research institute, Amsterdam, Netherlands

Contact
Dr. Joel J. Gagnier, jgagnier@umich.edu
Website: www.cosmin.nl

Table of Content

List of abbreviations	2
Cite & Funding	3
Introduction	4
General reporting recommendations relevant for all studies on measurement properties	5
Specific reporting recommendations for studies on Content Validity	8
Specific reporting recommendations for studies on Structural Validity	8
Specific reporting recommendations for studies on Internal Consistency	8
Specific reporting recommendations for studies on Cross-cultural Validity\Measurement Invariance	9
Specific reporting recommendations for studies on Reliability	10
Specific reporting recommendations for studies on Measurement error	10
Specific reporting recommendations for studies on Criterion Validity	11
Specific reporting recommendations for studies on Hypotheses Testing for Construct Validity	11
Specific reporting recommendations for studies on Responsiveness	11
References	13

List of abbreviations

CTT:	classical test theory
IRT/Rasch:	Item Response Theory and Rasch analyses
NA:	not applicable
Original CC:	original COSMIN checklist [1]
PROM:	patient-reported outcome measure
RoB:	Risk of Bias; it refers to the COSMIN Risk of Bias checklist [2]

Cite:

Joel J Gagnier, Jianyu Lai, Lidwine B Mokkink, Caroline B Terwee. COSMIN reporting guideline for studies on measurement properties of patient-reported outcome measures. Qual Life Res. 2021 Aug; 30(8):2197-2218. doi: 10.1007/s11136-021-02822-4.

Funding:

This study was funded by a Methods Grant from the Patient-Centered Outcomes Research Institute.

Introduction

The COSMIN Reporting Guideline is recommended for reporting on studies that evaluate the measurement properties of existing patient-reported outcome measures (PROMs). Adequate reporting of scientific research will increase the applicability of and contributions to scientific knowledge[3]. Studies examining measurement properties are often missing key information that may allow a reader of these studies (e.g., clinician, scientist, funder) to determine what methods were used, what the results are and ultimately what the research means for the evidence of the quality of a particular PROM. This reporting guideline can improve and direct the reporting of studies investigating any measurement properties of PROMs. Improving the reporting of these studies increases their transparency and therefore makes obvious their risk of bias as well as their import to scientific knowledge. This allows accurate methodological assessment of these papers, reliable application of their findings (e.g., to clinical research) and also allows researchers to build on or improve future investigations in the area.

The COSMIN Reporting Guideline was developed as a detailed and specific reporting guideline, for all sections of a manuscript and for all measurement properties that can be investigated within studies exploring the measurement properties of PROMs. More information on this study can be found here[4].

The guideline is a set of 71 items for inclusion in a reporting guideline for studies on measurement properties of PROMs. It contains 35 common recommendations to be used for all studies on any of the measurement properties, and 36 specific recommendations divided into reporting of the specific measurement properties.
The common recommendations are divided into items for reporting the title (n = 3 items), abstract (n = 7), introduction (n = 6), methods (n = 8), results (n = 3), discussion (n = 6), conclusions (n = 1), and other information (n = 1); the specific recommendations are divided into reporting items concerning content validity (n = 7), structural validity (n = 2), internal consistency (n = 3), cross-cultural validity\ measurement invariance (n = 5), reliability (n = 3), measurement error (n = 2), criterion validity (n = 3), hypotheses testing for construct validity (n = 5), responsiveness (n = 6).

We hope all relevant international scientific groups adopt these recommendations and that relevant peer-reviewed journals endorse and enforce them as they have for other reporting recommendations (e.g., CON SORT).

General Reporting recommendations relevant for all studies on measurement properties		
**Item Number**	**Item Name**	**Item Description**
**Report section: Title**		
T1	Patient Reported Outcome Measure (PROM)	The name of the PROM instrument(s) (and version if relevant) being studied
T2	Measurement Property (MP)	What MPs are being studied or more generally, that MPs are being studied (if there are many properties being investigated, for example)
T3	Study sample	General description of relevant study sample characteristics (e.g., condition of interest, language) and also any intervention or exposure (e.g., treatments) if applicable.
**Report section: Abstract**		
A1	PROM	The name of the PROM instrument(s) (and version if relevant) being studied (i.e. the SF-36 or SF-12; language version) or if it concerns an item bank (e.g., PROMIS instruments). The type of instrument (e.g. a self reported questionnaire or interview).
A2	Measurement Property	What MPs are being studied or more generally, that MPs are being studied (if there are many properties being investigated, for example)
A3	Design	The type of study being used to test the properties (e.g., test-retest design, longitudinal study, cohort, cross sectional, case series, randomized etc.). Other details of the study design if relevant (intervention/exposure, description of comparison instruments, outcomes other than PROMs).
A4	Sample	Inclusion / exclusion criteria. General description of relevant study sample characteristics (e.g., condition of interest, geographic location, language, other relevant demographic and baseline characteristics)
A5	Methods	A brief description of the methods for investigating each MP including statistical analyses
A6	Results	The main results for all MPs investigated reporting statistics for each result with measures of precision where appropriate.
A7	Discussion/Conclusions	A brief description of the results in the context of existing evidence, main strengths and drawbacks and the need for future research on the PROM(s) investigated.
**Report section: Introduction**		
I1	Name and describe the PROM of interest	Specify the name, type, language, and version of the PROM being investigated and how it was developed. Describe the construct the PROM aims to measure and its subscales; describe the structure of the PROM (e.g., the number of factors, the number of items, scoring algorithm); describe relevant instructions (like time period), and number or type of response categories. State whether the PROM is based on a reflective or formative model. Note: This information may also appear in the methods section in greater detail.
I2	Target population	Describe the specific target population that the PROM was designed for. The authors need to provide the appropriate and necessary characteristics of this population.
I3	Citation for the original development of the PROM	The citation for the original development paper(s) should be provided and other highly relevant citations related to the quality of the specific PROM under investigation.
I4	State of Knowledge & Rationale	A description of the current scientific knowledge (what is known) regarding the MPs of? the PROM under investigation. The authors should provide a literature review or refer to a recent review of all existing evidence of the specific version (e.g., language, short

		form) of the PROM and explain why the new study is necessary and important. The rational for the current proposed study should be given.
I5	Definitions	Specialized terms should be defined or explained.
I6	Objectives and Hypotheses	State the specific objective(s) of the research and hypotheses related to the specific PROM under investigation.
**Report section: General Methods**		
GM1	Study Design	State the key elements of the study design
GM2	Participants	State how the participants were chosen; the inclusion and exclusion criteria. (e.g., if a PROM for a specific condition, then the eligibility and selection criteria should reflect this).
GM3	PROM administration	An explicit description of how and when the PROM(s) were administered (e.g., in what setting) including data collection devices/system used (e.g. paper based, electronic administration / ePRO) should be provided.
GM4	Data collection procedures	Provide information about other data collection, exposure methods (e.g., allocation to interventions) and time points / follow-up points.
GM5	Power/sample size calculation	Provide a power calculation for all MP analyses. Alternatively, if a rule of thumb is used, state it and the source/citation.
GM6	Statistical analyses	Statistical analyses and tests corresponding to all hypotheses or objectives for all MPs should be reported. Where appropriate, a cut-off for statistical significance should be reported (e.g., p-value less than 0.05). A description of all statistics to be used to estimate the magnitude and direction of effect should also be reported, together with measures of variability or precision. Report statistical package used.
GM7	Missing data	State approaches or plan for dealing with missing data.
GM8	Post hoc analysis	The report should specify analyses that used data after the data collection period concluded (i.e., if the analyses were post hoc; secondary data analyses) and describe the rationale for any post hoc analyses.
**Report section: General Results**		
GR1	Missing data	The amount and reasons for missing data should be explained for all analyses for all PROMs (or other outcome measurement instruments) and relevant groups.
GR2	Participant/patient Characteristics	The study patients' characteristics should be described, including baseline PROM scores.
GR3	Sample size	If one study contained analyses using different sample sizes, the authors should report the sample size for each analysis.
**Report section: Discussion**		
D1	MP evidence	Per measurement property the authors should compare the result to the criteria for good measurement properties (e.g., COSMIN criteria)[27], and determine if the specific MP is sufficient or not. Note: This information may also appear in the results section in greater detail in a table for example.
D2	Practical relevance	The authors need to discuss the practical relevance of the findings.
D3	Strengths and limitations	Strengths and limitations of the study should be discussed. For example, discuss if there were any significant potential biases in the study that could have impacted the results.
D4	Generalizability	Generalizability issues related to the PROM results should be discussed. For example, discuss if the results could be generalized to other populations given the sample studied.
D5	Instrument changes	Discuss the need for modifications to the existing PROM or new

		PROM development. If you conclude that one of the measurement properties is insufficient, you could suggest some modification, or if it is really poor, you could suggest stopping use of the PROM (in the specific population or in general).
D6	Future Research	Report specifically the type of research needed to answer new questions arising out of these findings for the particular MP and PROM investigated.
**Report section: Conclusions**		
C1	Conclusions	State the overall conclusions for each MP and of the use PROM investigated.
**Report section: Other information**		
O1	Conflict of Interest	State any relevant conflict of interest related to the PROM under investigation (e.g., an author being the PROM developer, funding body etc).

Specific Reporting recommendations for studies on Content Validity		
**Item Number**	**Item Name**	**Item Description**
CV1	Relevance	Report if and how patients and/or professionals were asked whether each item is relevant for their experience with the condition
CV2	Comprehensiveness	Report if and how patients and/or professionals were asked whether all key concepts are included
CV3	Comprehensibility	Report if and how the comprehensibility of the PROM instructions, items, response options, and recall period was assessed
CV4	Relevance results	Report if all items were considered relevant for the construct, population, and context of use of interest by patients and/or professionals
CV5	Response options and recall period	Report whether the response options and recall period were considered appropriate by patients and/or professionals
CV6	Comprehensiveness results	Report whether patients and/or professionals considered all key concepts to be included in the PROM
CV7	Comprehensibility results	Report whether patients understood the PROM instructions, items, and response options as intended

Specific Reporting recommendations for studies on Structural Validity		
**Item Number**	**Item Name**	**Item Description**
SV1	Factor Analyses: Classical Test Theory (CTT) PROMs	Report details of the methods and results for any exploratory or confirmatory factor analyses. State the rational for any explorative factor analyses (e.g., no clear a priori hypotheses). For CFA, describe and justify the factor structure of tested models. Methods and results for checking of the assumptions should be described, the method of estimation, goodness-of-fit statistics and cut-off points for good model fit, including factor loadings of best-fitting model.
SV2	Item Response Theory (IRT) analyses	Type of IRT/Rasch model should be reported. Also report the method of estimation, methods and results for checking of the assumptions (unidimensionality (see factor analysis), local dependency (e.g., residual correlations), monotonicity; (e.g. Mokken scaling), goodness-of-fit statistics, and cut-off points for goodness of item/model fit, and all item parameters.

Specific Reporting recommendations for studies on Internal Consistency		
**Item Number**	**Item Name**	**Item Description**
IC1	Unit of measurement	Report internal consistency methods and results for each unidimensional scale or subscale. Report all evidence or assumptions associated with unidimensionality.
IC2	Continuous scores	Report Cronbach’s alpha or omega statistics. Report other statistics calculated for internal consistency of continuous scores.
IC3	Dichotomous scores	Report Cronbach’s alpha or Kuder-Richardson coefficient. Report other statistics calculated for internal consistency of dichotomous scores.

Specific Reporting recommendations for studies on Cross-Cultural Validity\Measurement Invariance		
**Item Number**	**Item Name**	**Item Description**
CCV1	Comparator Group(s)	Report characteristics of (sub)groups being compared. Include sample sizes in each group.
CCV2	Factor Analyses: Classical Test Theory (CTT) PROMs	Report details of the methods and results for multiple-group confirmatory factor analyses, logistic regression analyses, or other analyses performed. Describe and justify the series of tested models, including constraints of factor loadings, intercepts and variances in CFA. Methods and results for checking of the assumptions should be described. criteria to define invariance. Describe the method of estimation, goodness-of-fit statistics and criteria used to flag items for measurement invariance.
CCV3	Item Response Theory (IRT) analyses	Type of IRT/Rasch model should be reported. Also report the methods and results for checking of the assumptions (unidimensionality (see factor analysis), local dependency (e.g., residual correlations), monotonicity; (e.g. Mokken scaling).. Describe statistical packages, method of estimation, criteria used to flag items for DIF, and methods and results of all model comparisons.

Specific Reporting recommendations for studies on Reliability		
**Item Number**	**Item Name**	**Item Description**
R1	PROM Administrations	Report the total number of measurements made and if the measurements were applied to the same samples using the same PROM. The process of administrating the measurements to the patients should be described, including who administered it (i.e., did the patient complete it or was there a proxy), when, how and any time intervals between administrations should be reported. This should include: time interval between repeated measurements (e.g., was the patient stable or not), the test type (e.g. a self-administered questionnaire, an interview-based PROM), the setting in which the instrument was administered (e.g., at the hospital, or at home), and the instructions given for completing it. If relevant, other instruments or measurements accompanying the repeated PROM measurement. Also, if relevant, the independence (whether the PROM was completed without knowledge of the previous scores) of the administrations.
R2	Statistical analyses	All statistical analyses and results specific to the reliability assessment(s) should be described and their use justified (e.g., the intraclass correlation coefficient (ICC) model or type of Kappa coefficient used). Also, describe the variance components, and the weighting scheme used for ordinal scores (e.g., linear or quadratic weights).
R3	Methods to improve reliability	Report any methods used to improve reliability such as restriction of the sample, training of researchers and standardization of methods, and averaging of repeated measurements.

Specific Reporting recommendations for studies on Measurement Error		
**Item Number**	**Item Name**	**Item Description**
ME1	PROM administrations	Report the total number of measurements made and if the measurements were applied to the same samples using the same PROM. The process of administrating the measurements to the patients should be described, including who administered it (i.e., did the patient complete it or was there a proxy), when, how and any time intervals between administrations should be reported. This should include: time interval between repeated measurements (e.g., was the patient stable or not), the test type (e.g. a self-administered questionnaire, an interview-based PROM), the setting in which the instrument was administered (e.g., at the hospital, or at home), and the instructions given for completing it. If relevant, other instruments or measurements accompanying the repeated PROM measurement. Also, if relevant, the independence (whether the PROM was completed without knowledge of the previous completion) of the administrations.
ME2	Statistical analyses	All statistical analyses and results specific to measurement error assessment(s) should be described and their use justified. Specifically, for continuous scores report the Standard Error of Measurement (SEM; Specify the exact model used to calculate the SEM (i.e., SEM consistency or SEM agreement)), Smallest Detectable Change (SDC; specify formula used, included the model of the SEM when based on the SEM) or Limits of Agreement (LoA). For dichotomous/nominal/ordinal scores report marginals (raw data) and the percentage specific (e.g. positive and negative) agreement.

Specific Reporting recommendations for studies on Criterion Validity		
**Item Number**	**Item Name**	**Item Description**
CriV1	Criterion	Report the details of the criterion used and why it was used. Justification of the gold standard must be reported. Also, describe (if applicable) how and why the criterion was dichotomized or classified. Also, how and when the criterion was administered (e.g., if independent from the PROM).
CriV2	Continuous scores	Report correlations (when criterion has continuous scores) or the area under the receiver operating characteristic (ROC) curve (when criterion is dichotomous).
CriV3	Categorical scores	Described how (and why) the PROM was dichotomized or made into multiple categories. Report sensitivity and specificity statistics.

Specific Reporting recommendations for studies on Hypotheses Testing for Construct Validity		
**Item Number**	**Item Name**	**Item Description**
ConV1	Comparator instrument(s)	The comparator instruments should be appropriately described in terms of the construct(s) they intend to measure. Report the measurement properties of the comparator instruments and related citations or data.
ConV2	Comparator Group(s)	Report characteristics of groups being compared. Include sample sizes in each group.
ConV3	Hypotheses	Report all hypotheses including the direction and magnitude of the expected correlations between the PROM of interest and another measurement instrument, or the direction and magnitude of differences in scores of the PROM between groups.
ConV4	Statistical analyses	Report all statistical methods and results used to test each hypothesis.
ConV5	Results	Report which specific results are in accordance with its hypothesis.

Specific Reporting recommendations for studies on Responsiveness		
**Item Number**	**Item Name**	**Item Description**
Resp1	Comparison Instrument(s)	The comparator instruments should be appropriately described in terms of the construct(s) they intend to measure. Report the measurement properties of the comparator instruments and related citations or data.
Resp2	Comparator Group(s)	Report characteristics of groups being compared. Include sample sizes in each group.
Resp3	Hypotheses	Report all hypotheses including the direction and magnitude of the expected correlations between changes in the PROM of interest and change in another measurement instrument, or the direction and magnitude of differences in change scores of the PROM between groups.
Resp4	Measurement procedures	Report if measurements were applied to the same sample using the same instruments. Describe the measurement procedures, including time intervals between different measurement instruments.
Resp5	Interim period	The interim period between time points should be described.
Resp6	Intervention/Exposure	Describe the intervention given or exposure in the interim period if relevant.
Resp7	Patients changed	Report the proportion of patients that improved or deteriorated (and the details of any anchor used) on the construct measured on all PROMs. Report any changes in

		scores of the PROM in the target population for the research application relative to the predefined hypotheses
Resp8	Statistical analyses	Report all statistical methods and results used to test each hypothesis.
Resp9	Results	Report which specific results are in accordance with its hypothesis.

References

1. Mokkink LB, Terwee CB, Patrick DL, et al. The COSMIN checklist for assessing the methodological quality of studies on measurement properties of health status measurement instruments: an international Delphi study. *Qual Life Res* 2010;19(4):539-49. doi: 10.1007/s11136-010-9606-8 [doi]
2. Mokkink LB, de Vet HCW, Prinsen CAC, et al. COSMIN Risk of Bias checklist for systematic reviews of Patient-Reported Outcome Measures. *Qual Life Res* 2018;27(5):1171-79. doi: 10.1007/s11136-017-1765-4
3. Ioannidis JP. How to make more published research true. *PLoS Med* 2014;11(10):e1001747. doi: 10.1371/journal.pmed.1001747 [published Online First: 2014/10/22]
4. Gagnier JJ, Lai J, Mokkink LB, et al. COSMIN reporting guideline for studies on measurement properties of patient-reported outcome measures. *Qual Life Res* 2021 doi: 10.1007/s11136-021-02822-4 [published Online First: 2021/04/06]
